# Validation and measurement invariance of the Compulsive Exercise Test among Brazilian and American young adults

**DOI:** 10.1007/s40519-023-01627-3

**Published:** 2024-01-03

**Authors:** Priscila Figueiredo Campos, Leslie D. Frazier, Maurício Almeida, Pedro Henrique Berbert de Carvalho

**Affiliations:** 1 NICTA, Body Image and Eating Disorders Research Group, Federal University of Juiz de Fora, 745 São Paulo Street, City Center, Governador Valadares, Minas Gerais 35010-150 Brazil; 2 Department of Psychology, Florida International University, Miami, FL USA; 3 AMBULIM, Eating Disorders Program, Institute of Psychiatry, University of São Paulo, São Paulo, Brazil

**Keywords:** Exercise dependence, Pathological exercise, Disordered eating, Eating disorders, Psychometrics, Cross-cultural study

## Abstract

**Purpose:**

To evaluate compulsive exercise, researchers often rely on the widely used Compulsive Exercise Test (CET). However, the measure has shown unstable factor structure in several validation studies and is not available in Portuguese for use in Brazil. We aimed to describe the translation and cultural adaptation of the CET to Brazilian Portuguese, to test several factor structures among Brazilian and US samples of men and women, to test measurement invariance across countries, and to evaluate its internal consistency. Furthermore, we sought to evaluate convergent validity, correlating the CET with a measure of eating disorder symptoms, and to compare compulsive exercise symptoms between countries.

**Methods:**

Four models of the latent structure of the CET were tested using confirmatory factor analyses (CFAs), three-factor structure with 15 items, three-factor structure with 18 items, four-factor structure with 21 items, and the original five-factor structure with 24 items, in a sample of 1,531 young adults (601 Brazilians and 930 Americans), aged 18–35 years.

**Results:**

A series of CFAs demonstrated that the three-factor structure with 15 items showed a better fit to the data. This model demonstrated good convergent validity and internal consistency. Results from the CET multigroup CFA showed evidence for the invariance at the configural, metric, and scalar levels across Brazilians and Americans. Furthermore, significant differences were found between Brazilians and Americans, with Brazilians demonstrating higher scores on the *Avoidance and rule-driven behavior* and *Mood improvement* subscales, whereas US participants scored higher on the *Weight control exercise* subscale.

**Conclusions:**

Results support the three-factor structure with 15 items to be used as a measure of compulsive exercise among Brazilians and Americans, allowing cross-cultural comparisons between these countries.

**Level of evidence:**

Level V, Cross-sectional, Psychometric study.

## Introduction

Dysfunctional exercise encompasses behaviors or exercise routines considered harmful to health, such as exercise dependence, compulsive exercise, excessive exercise, exercise addiction [1–5]. Compulsive exercise can be understood as a driven behavior that is not easily interrupted or reduced [6]. It is not just uncontrolled exercise behaviors that classify compulsive exercise, but the motivation for which it is practiced [7]. It is believed that dissatisfaction with body weight and shape are the biggest motivators for maintaining exercise in compulsive exercise [6]. The compulsive exercise model proposed by Meyer and colleagues [6] suggests compulsive exercise as a condition characterized by an association of concerns about weight and body shape and continued engagement in order to alleviate extreme guilt and/or negative affect when exercise is carried out, and to avoid exercise withdrawal.

A recent systematic review identified 17 different dysfunctional exercise screening measures [8]. Of these, only six were classified as measures of compulsive exercise behaviors. These are: the Obligatory Exercise Questionnaire (OEQ) [9] and its later versions [10–12], the Excessive Exercise Scale (EES) [13], and the Compulsive Exercise Test (CET) [14].

Compulsive exercise has been proposed as one of the maladaptive elements that may relate to the development and maintenance an eating disorder [15]. Research shows that up to 80% of individuals with anorexia nervosa and 40% of individuals with bulimia nervosa report dysfunctional exercise behaviors [15–17]. Given the strong association between compulsive exercise and eating disorders in clinical and epidemiological data [15, 18], the CET has been widely used [19] to assess compulsive exercise behaviors. The measure was developed to assess the main factors for compulsive exercise, given the lack of an instrument that was sensitive to issues of connection between eating disorders and dysfunctional exercise [14]. Most of the available dysfunctional exercise measures were developed based on their addictive characteristics [8].

### The Compulsive Exercise Test (CET)

The CET [14] is a measure of compulsive exercise composed of 24 items. Through principal component analysis with oblique oblimin rotation, Taranis et al. [14] found a five-factor structure: *Avoidance and rule-driven behavior* (items 9, 10, 11, 15, 16, 20, 22, and 23), *Weight control exercise* (items 2, 6, 8, 13, and 18), *Mood improvement* (items 1, 4, 14, 17, and 24), *Lack of exercise enjoyment* (items 5, 12, and 21), and *Exercise rigidity* (items 3, 7, and 19). The CET showed good internal consistency, content validity, and concurrent validity among university women from the United Kingdom and Australia [14]. The CET also demonstrated strong positive associations with a measure of eating disorders pathology (the Eating Disorder Inventory) [20] and another measure of dysfunctional exercise (the Commitment to Exercise Scale) [21].

The CET has been tested in athletes [22], adolescents [23–25], regular exercisers [26], university students [27], and adults diagnosed with eating disorders [4, 28–31]. It is worth noting that the original five factors structure with 24 items has rarely been confirmed [22, 26, 27]. In fact, only Goodwin et al. [24] was able to reproduce the original factorial structure. However, it must be noted that both Taranis et al. [14] and Goodwin et al. [24] performed principal component analysis (PCA) instead of factor analysis, and that the original factor structure was not confirmed through subsequent analysis (e.g., confirmatory factor analysis—CFA).

Principal component analysis (PCA) is a statistical method that aims to reduce the number of variables observed in “principal components,” while retaining as much variance as possible from the original item, while considering both common and specific variance [32, 33]. On the other hand, exploratory factor analysis (EFA) identifies the latent constructs and the underlying factor structure of a set of variables while taking into account only the common variance [32, 33]. Although both techniques have similarities, and in some cases, present similar results, they have different purposes [34].

Furthermore, during the development of the CET, Taranis et al. [14] found that the *Lack of exercise enjoyment* subscale did not correlate with the CET total score (*r* = 0.10, *p* > 0.05), indicating that this subscale was perhaps not significant as a measure of compulsive exercise. In fact, Plateau et al. [22] tested the CET among athletes finding a three-factor structure with 15 items after excluding all items from the *Lack of exercise enjoyment* and *Exercise rigidity* subscales. Rica et al. [27] also found good adjustment indices using the structure proposed by Plateau et al. [22] in a Spanish sample of university students. Interestingly, the study by Goodwin et al. [24] also highlights some concerns regarding the CET items. For example, item #11 (“*I usually continue to exercise despite injury or illness, unless I am very ill or too injured*”) and item #15 (“*If I miss an exercise session, I will try and make up for it when I next exercise*”)—from the *Avoidance and rule-driven behavior subscale*—showed low factor loadings. Low factor loadings were also found for eight items in the study of Vrabel and Bratland-Sanda [30] among adults diagnosed with eating disorders. Despite that, both Goodwin et al. [24] and Vrabel and Bratland-Sanda [30] chose to keep problematic items, which is a concern according to some guidelines [34].

Given the lack of consensus of the best factor structure of the CET, Limburg et al. [26] proposed to analyze and compare several different structures of the instrument (i.e., one-factor solution with a total of 18, 21, or 24 items; three-factors with a total of 18 items; four-factors with a total of 21 items; and a five-factor structure with a total of 24 items). Although the authors claim that all structures tested are adequate [26], most of the adjustment fit indices found were below recommended and acceptable values [35]. In fact, results show that the three-factor solution with 18 items (i.e., excluding the *Lack of exercise enjoyment* and *Exercise rigidity* subscales) was the most parsimonious one [26]. Although, Plateau et al. [22] and Limburg's [26] had found a three-factor solution, Plateau et al. [22] chose to exclude items that did not present adequate factor loading (i.e., items #8, #11 and #15).

Interestingly, Schlegl et al. [29] found a better fit of four-factor [26] and three-factor [22] solutions compared to the original five-factor model [14] in a large clinical sample of adolescent and adult inpatients with anorexia nervosa and bulimia nervosa. These authors also tested measurement invariance of the CET across diagnosis and age group. Not surprisingly, results show that only three subscales were satisfactorily invariant to measurement (i.e., *Avoidance and rule-driven behavior*, *Weight control exercise*, and *Mood improvement* subscales), but not the CET total score [29].

Measurement invariance of the CET was also evaluated across athletes performing at competitive levels [27]. However, the invariance of the CET across different linguistic and cultural contexts had not been explored. Given the results, it was recommended to further explore and/or revise the original CET, including investigation in other samples.

Additionally, it’s worth noting that the CET has been applied in clinical [4, 28–31] and non-clinical samples [14, 22–27]. When applied in clinical samples with ED the factors driving compulsive exercise differ according ED diagnostic subtype, so this should be taken into account during ED treatment [28]. In non-clinical samples, sensitivity analysis is necessary to assess patients with high risk factor for compulsive exercise and ED. Given all the concerns regarding the factorial structure of the CET, many researchers choose to use its factors in isolation, not the total score [22, 26].

### The present study

Given the lack of stability of the CET factorial structure described in the literature, as well as the lack of a Brazilian Portuguese version of the CET, the objectives of this research were: (a) to examine the factor structure (i.e., CFAs) of previous CET models [14, 22, 26]; (b) to evaluate the convergent validity of the CET with a measure of eating disorder symptoms, and (c) to estimate the internal consistency of the CET scores. Furthermore, the present study sought to evaluate measurement invariance of the CET among Brazilians and a comparable sample of U.S. adults, with the aim of examining comparative profiles of compulsive exercise across different cultures. Finally, if invariance is assumed, comparison between U.S. and Brazilian samples will be conducted to test differences between Brazilians and Americans along to the CET total score and its subscales.

The choice for comparing adults from Brazil and the United States of America (USA) is deliberate. The U.S. is the global leader, as the country with the highest number of gyms/fitness facilities, followed by Brazil [36], showing that both countries have comparatively greater interest in physical exercise. The U.S. and Brazil also share the distinction of having the highest number of practices for aesthetic plastic surgeries [37] and the highest use of anabolic steroids [38]—all of which are common body-change strategies that may relate to body weight, shape, and appearance concerns. It is known that greater physical appearance concern can lead individuals to develop some behaviors that are harmful to health. Not surprisingly, both countries have a high frequency of people with body dissatisfaction and disordered eating [39].

In the present study we hypothesized that the CET three-factor solution with 15 items (i.e., *Avoidance and rule-driven behavior*, *Weight control exercise*, and *Mood improvement*) proposed by Plateau et al. [22] would present the best fit to the data (Hypothesis 1). We expected the CET subscales to be positively associated with a measure of eating disorder symptoms (Hypothesis 2), as found in previous studies [15, 22, 24–26]. We also hypothesized that the CET subscales would demonstrate adequate internal consistency (Hypothesis 3). Furthermore, we anticipated that the CET would show measurement invariance across Brazilians and Americans (Hypothesis 4). Finally, we expected the Brazilians and Americans would present similar scores of the CET subscales (Hypothesis 5).

## Materials and methods

### Ethical statement

This study is part of a larger cross-sectional, cross-cultural study conducted in Brazil and the U.S. that aimed to evaluate body image concerns, eating disorders, compulsive exercise, and psychological functioning. Ethics approval was obtained from each relevant Institutional Review Board. All procedures are in accordance with the principles specified in the Declaration of Helsinki. The study was conducted and data gathered consistent with the American Psychological Associations Journal Article Reporting Standards for Quantitative Data (JARS).

### Participants

The total sample consisted of 1,531 young adults (601 Brazilians and 930 Americans), aged 18 and 35 years. The inclusion criteria were age and self-reported engagement in regular physical exercises. Exclusion criteria were not completing the full research protocol. The sample size exceeds the minimum sample size required for factor analyses (i.e., 10 people per item) [34].

### Procedures

After ethics approval, the first step consisted of contacting the first author of the original CET validation study, who consented to our study. The translation, back-translation and cross-cultural adaptation of the CET for the Brazilian Portuguese were conducted according to standardized international recommendations [34]. Semantic, idiomatic, cultural, conceptual, and operational equivalence analyses were performed following the Herdman et al. (1998) proposal. Equivalences between the original and Brazilian versions were analyzed by an expert committee (i.e., the researchers responsible for the study, a language expert, a translator, and two exercise dependence specialists). Verbal understanding of the items and instructions for completing the scale were verified using two focus groups (one for each sex). All necessary adjustments were made, and the final instrument was rechecked by the expert committee. The cross-culturally adapted Brazilian version of the CET was used in the following study’s phases.

The study was advertised on social media, online forums, email lists, and communities. Potential participants received information about the research, and if interested, received a URL to the study. All participants were consented, and once consented, they were taken to a fully online survey was hosted on Qualtrics^®^ platform and Internet Protocol (IP) addresses were recorded to ensure that no participant completed the survey more than once. The research was completely anonymous, the data were treated confidentially, and all participants participated voluntarily and were not remunerated or subsidized for their participation. In the U.S. sample, participants received one extra-credit research point for participation.

### Measures

#### Demographics

All participants were asked to report their age, race/ethnicity, sex, gender identity, sexual orientation, education, and income. In addition, they were asked about meal preparation, health status, and presence of a diagnosis of illness or eating disorders.

#### Compulsive Exercise Test (CET)

The CET [15] is a self-report measure of compulsive exercise, composed of 24 items presented on a 6-point Likert-type scale, ranging from 0 (*never true*) to 5 (*always true*). The score of each of the CET subscales (i.e., *Avoidance and rule-driven behavior*, *Weight control exercise*, *Mood improvement*, *Lack of exercise enjoyment*, *Exercise rigidity*) are obtained by summing their items. The higher the score, the greater the compulsive exercise. The original English version of the CET (i.e., in English) was administered to the U. S. sample, and the cross-culturally adapted Portuguese version was administered to the Brazilian sample.

#### Eating disorder examination questionnaire (EDE-Q)

The EDE-Q is a 28-items questionnaire that assesses attitudes related to key features of eating disorders psychopathology over a 28-day period [40]. The original English version was used for the U.S. sample. The cross-culturally adapted Portuguese version of the EDE-Q [41] was administered for Brazilian sample. The EDE-Q presents good indicators of validity in both countries [40, 42]. The EDE-Q’s 22 items are rated on a 7-point Likert scale, ranging from 0 (*Never* or *Not at all*) to 6 (*Every day* or *Markedly*). The global score is obtained by averaging items’ scores. Higher scores indicate greater eating disorder symptom severity. The behavioral frequency items (i.e., items 13–18) were not included in the analyses. In the present study, the EDE-Q revealed good internal consistency for both the Brazilians (McDonald’s *ω* = 0.94 [95% CI = 0.93, 0.94]) and the Americans (*ω* = 0.95 [95% CI = 0.94, 0.96]).

### Data analysis

As suggested by Parent [43], 420 Americans (31.1%) and 72 Brazilians (10.7%) were excluded because they had more than 80% missing data. Responses from remaining participants were inspected and showed to be consistent with Missing Completely at Random (χ^2^(79) = 95.432, *p* = 0.10) using Little's test [42]. We imputed missing values (4.7%; n = 68) using expectation–maximization method [43].

Descriptive statistics were performed for numerical data (means and standard deviations) and categorical data (absolute and relative frequencies). The items in the Brazilian and American versions of the CET were inspected for univariate normality. Skewness (< 3) and kurtosis (< 10) values, and multivariate normality (Mardia coefficients < 5) were considered adequate [44]. Multivariate outliers were inspected using Mahalanobis distance squared (*D*^2^). All analyzes were performed with JASP v. 0.16.4.0 [45] and a significance level of 5% (*p* < 0.05) was adopted.

#### Confirmatory factor analysis (CFA)

The weighted least square mean and variance adjusted (WLSMV) method was used to estimate the models for the Brazilian and American samples [44]. A total of four models were tested: (a) the original five-factor solution with 24 items [15]; (b) a three-factor solution with 15 items [22]; (c) a three-factor solution with 18 items [26]; and (d) a four-factor solution with 21 items [26].

The adequacy of the models was evaluated by using the chi-square corrected by degrees of freedom (χ^2^/*df*) and by multiple adjustment indexes: comparative fit index (CFI), Tucker-Lewis index (TLI), root mean squared error approximation (RMSEA), and standardized root mean square (SRMR) [35]. The following values are considered acceptable: χ^2^/*df* (≤ 5), CFI and TLI (close to 0.95), RMSEA (< 0.08; [95% confidence interval, *p* > 0.05]), and SRMR (< 0.08). The factor loading (*λ*) was analyzed, and a cutoff point of 0.50 was considered adequate [44].

#### Convergent validity

To analyze the evidence of convergent validity, the scores of the CET models and their factors were correlated with eating disorder symptoms (EDE-Q global score). The analyses were conducted using Spearman’s rank order correlation coefficient (*r*_*s*_). Correlations of 0.20, 0.40 and 0.60 were considered small, moderate, and large, respectively [44].

#### Internal consistency

The estimated internal consistency of the instruments applied in the present study was evaluated using McDonald's *ω*. Values of *ω* close to 0.70 were considered acceptable [46].

### Measurement invariance

Measurement invariance analysis for the most parsimonious CET factor model (i.e., the three-factor solution with 15 items) [22] was conducted using the Brazilian and American samples to evaluate configural (assessing whether the hypothesized factor structure fits well across different samples), metric (assessing whether the factor loadings are equivalent across the samples), and scalar (assessing whether the item intercepts are equivalent across the samples) invarianceΔCFI < 0.005, ΔRMSEA < 0.010, and ΔSRMR < 0.025 were considered indicators of metric invariance, and scalar invariance was supported when ΔCFI < 0.005, ΔRMSEA < 0.010, and ΔSRMR < 0.005 [47, 48].

### Comparison between U.S. and Brazilian samples

Multivariate analysis of covariance (MANCOVA) was performed to compare study variables in the two groups. Considering that age can influence the compulsive exercise behaviors [3], it was included as a covariate. The coefficient Partial *n*^*2*^ was used to report effect size for variables with 0.10–0.29 being considered small, 0.30–0.49 being considered medium, and ≥ 0.50 being considered large [49].

## Results

### Descriptive analysis

The sociodemographic characteristics of the two samples are shown in Table 1.

**Table 1:** Descriptive Statistics and Test of Differences between the Brazilian and American Samples

Variables^a^	Brazilians (n = 601)	Americans (n = 930)	Comparison tests^b^	Effect size^c^
	*M* (*SD*)	*M* (*SD*)		
Age (years)	23.62 (4.51)	22.35 (3.64)	*t*(1528) = 6.61; *p* < 0.001	*d* = 0.35
Sexual identity	N (%)	N (%)		
Male	208 (34.6)	120 (12.9)	χ^2^(2) = 150; *p* < 0.001	*V* = 0.32
Female	336 (56.1)	781 (83.8)		
Other	57 (9.3)	29 (3.3)		
Sexual orientation				
Heterosexual	443 (74.1)	746 (80.4)	χ^2^(3) = 11.9; *p* = 0.007	*V* = 0.08
Homosexual	29 (4.8)	29 (3.1)		
Bisexual	99 (16.6)	107 (11.5)		
Other	30 (4.5)	48 (5.0)		
Eating disorders				
Anorexia	3 (0.6)	15 (2.5)	χ^2^(4) = 91.3; *p* < .001	*V* = 0.24
Bulimia	7 (1.4)	6 (1.1)		
Binge Eating Disorder	24 (4.7)	9 (1.5)		
Any other Eating Disorder	11 (2.2)	4 (0.6)		
There is no Eating Disorder	556 (91.1)	896 (94.3)		

*M* mean, *SD* standard deviation, *N* absolute frequency, *%* relative frequency (percentage); ^a^Self-reported information, ^b^Comparison tests were conducted using *t* test for continuous variable (age) and chi-square test for categorical variables (sexual identity, sexual orientation, and eating disorders). ^c^Effect sizes are described by Cohen’s *d* or Cramer’s *V*

### Confirmatory factor analysis (CFA)

Normality violation indicators were not identified after inspection of univariate and multivariate normality. Multivariate outliers (*D*^2^) were also not identified. Table 2 presents a comparison of the fit indices of the tested models based on estimation techniques.

**Table 2:** Fit Indexes for the Compulsive Exercise Test Models in Confirmatory Factor Analysis

Model	χ^2^/*df*	CFI	TLI	RMSEA (95% CI)	SRMR	*λ*
Taranis et al. (2011) 5 factors with 24 items						
Brazilian sample	5.88	0.87	0.85	0.09 (0.08–0.09)	0.11	0.17–10.05
American sample	9.09	0.85	0.83	0.09 (0.09–0.09)	0.09	0.33–0.98
Plateau et al. (2014) 3 factors with 15 items						
Brazilian sample	2.93	0.99	0.98	0.06 (0.05–0.06)	0.06	0.64–0.86
American sample	4.40	0.98	0.98	0.06 (0.05–0.07)	0.06	0.61–0.83
Limburg et al. (2019) 3 factors with 18 items						
Brazilian sample	3.85	0.97	0.97	0.06 (0.06–0.07)	0.07	0.06–0.87
American sample	6.02	0.97	0.96	0.07 (0.07–0.08)	0.07	0.13–0.85
Limburg et al. (2019) 4 factors with 21 items						
Brazilian sample	5.61	0.97	0.96	0.07 (0.07–0.08)	0.07	0.03–0.86
American sample	5.62	0.97	0.96	0.07 (0.06–0.07)	0.07	0.07–0.81

*χ*^*2*^*/df* Chi-square corrected by degrees of freedom (≤ 5), *CFI* comparative fit index (close to 0.95), *TLI* Tucker–Lewis index (close to 0.95), *RMSEA* root-mean-square error of approximation, *95% CI* 95% of confidence interval (< 0.08; [95% confidence interval, *p* > 0.05]), *SRMR* standardized root-mean-square residual (< 0.08), *λ* factor loading

As can be seen in Table 2, the original five-factor solution [15] did not show good fit indices in any of our samples. Values of χ^2^/*df* were high and CFI and TLI indices were very low. Moreover, items #5 (“*I find exercise a chore*”) and #8 (“*I do not exercise to be slim*”) had a factorial load below the adopted cut-off point (i.e., *λ* ≥ 0.50; [44]). The four-factor solution [26] did not show adequate indices for both samples due to high χ^2^/*df* values and low factor loading on some items. The three-factor solution with 18 items [26] did not show adequate indices for the American sample due to high χ^2^/*df* value and low factor loading on item #8. The most parsimonious model was the three-factor solution with 15 items [22], which showed good fit indices for both samples. All items presented high factor loadings (*λ* > 0.61).

### Convergent validity

Table 3 shows evidence of convergent validity between the CET subscales and the EDE-Q global score. As expected, the CET subscales showed significant correlations with the EDE-Q, except for the *Mood Improvement* subscale, which did not show a significant correlation with the EDE-Q among Brazilians. Small and significant correlations were observed between the EDE-Q and the *Avoidance and rule-driven behavior* subscale in both samples. The *Weight control exercise* subscale showed a strong correlation with the EDE-Q in both samples. Finally, a small correlation was observed between the *Mood improvement* subscale and EDE-Q among Americans.

**Table 3:** Bivariate Correlations (Spearman Rank Order Correlation) between the CET Subscales and Disordered Eating Behaviors (EDE-Q global score) for Brazilian (N = 601) and American (N = 930) Samples

	CET_Avoidance_	CET_Weight_	CET_Mood_	EDE-Q_Global score_
CET_Avoidance_	–	0.57*	0.49*	0.39*
CET_Weight_	0.53*	–	0.43*	0.61*
CET_Mood_	0.54*	0.31*	–	0.15*
EDE-Q	0.32*	0.63*	0.04	–

Below the diagonal center line are data from the Brazilian sample; above the diagonal center line are the American sample data; *CET* Compulsive Exercise Test, *CET*_*Avoidance*_ Subscale Avoidance and rule-driven behavior, *CET*_*Weight*_ Subscale Weight control exercise, *CET*_*Mood*_ Subscale Mood improvement, *EDE-Q* Eating Disorder Examination Questionnaire (global score)

*p < 0.01

### Internal consistency

In the total sample (i.e., Brazilians and Americans), the CET subscales demonstrated a good internal consistency (i.e., the *Avoidance and rule-driven behavior* (*ω* = 0.91 [95% CI = 0.90, 0.92]), the *Weight control exercise* (*ω* = 0.82 [95% CI = 0.80, 0.83]), and the *Mood Improvement* (*ω* = 0.87 [95% CI = 0.86, 0.88])). Similarly, in separate samples of Brazilians and Americans, the CET subscales also showed good internal consistency (i.e., the *Avoidance and rule-driven behavior* (Brazilians: *ω* = 0.92 [95% CI = 0.91, 0.93]; Americans: *ω* = 0.90 [95% CI = 0.89, 0.91]), the *Weight control exercise* (Brazilians: *ω* = 0.82 [95% CI = 0.79, 0.84]; Americans: *ω* = 0.81 [95% CI = 0.79, 0.83]), and the *Mood Improvement* (Brazilians: *ω* = 0.90 [95% CI = 0.89, 0.91]; Americans: *ω* = 0.84 [95% CI = 0.83, 0.86]). Taken together, these results provide support for the internal consistency reliability of the CET.

### Measurement invariance

Results from the CET multigroup CFA showed evidence for the invariance at the configural level, indicating that the factorial structure is the same across Brazilians and Americans (Table 4). In addition, metric (ΔCFI = 0.002, ΔRMSEA = − 0.002, ΔSRMR = − 0.003) and scalar (ΔCFI = 0.002, ΔRMSEA = − 0.001, ΔSRMR = 0.001) invariance were supported.

**Table 4:** Measurement Invariance of the CET between Brazilian (N = 601) and American (N = 930) Samples

Fit	χ^2^	*df*	*p* value	CFI	ΔCFI	RMSEA	ΔRMSEA	SRMR	ΔSRMR
Configual	638.102	174	< 0.001**	0.983	–	0.059	–	0.063	–
Metric	714.420	186	< 0.001**	0.981	0.002	0.061	− 0.002	0.066	− 0.003
Scalar	771.035	198	< 0.001**	0.979	0.002	0.062	− 0.001	0.065	0.001

*Fit Configural* base model, *Fit Metric* testing for differences in factor structure, *Fit Scalar* testing for differences in item means; *χ*^*2*^ Chi-square, *df* degrees of freedom, *CFI* comparative fit index, *RMSEA* root mean square error of approximation, *SRMR* standardized root-mean-square residual, Metric cut-off (ΔCFI < 0.005, ΔRMSEA < 0.010, and ΔSRMR < 0.025), Scalar cut-off (ΔCFI < 0.005, ΔRMSEA < 0.010, and ΔSRMR < 0.005)

**p < 0.001

### Comparison between U.S. and Brazilian samples

The MANCOVA results (Pillai's trace = 0.07, *f* [3, 1525] = 38.5, *p* ≤ 0.001) indicate that there are significant differences among the countries regarding CET subscales (see Table 5). Brazilians scores higher on the *Avoidance and rule-driven behavior* and *Mood improvement* subscales; whereas Americans scored higher on the *Weight control exercise* subscale. In all comparisons the effect size was small.

**Table 5:** MANCOVA Results for Brazil *versus* United States of America (USA) on the CET Subscales Controlling for Age

Variables	Brazil	EUA	f	df	p-value	Partial n^2^
	M (SD)	M (SD)				
CET_Total_	50.0 (16.8)	48.6 (16.0)	2.73	1	0.098	0.005
CET_Avoidance_	15.8 (8.27)	14.7 (7.47)	7.73	1	0.006*	0.005
CET_Weight_	12.5 (5.45)	14.1 (5.36)	32.19	1	0.001**	0.021
CET_Mood_	21.7 (7.11)	19.8 (6.68)	27.62	1	0.001**	0.018

*M* mean, *SD* standard deviation, *f* MANCOVA test, *df* degrees of freedom, *CET* Compulsive Exercise Test, *CET*_*Avoidance*_ Subscale Avoidance and rule-driven behavior, *CET*_*Weight*_ Subscale Weight control exercise, *CET*_*Mood*_ Subscale Mood improvement

^*^p < 0.01, **p < 0.001

## Discussion

The number of studies on dysfunctional exercise has grown substantially in recent years. Thus, there is an increasing concern to develop/validate valid and reliable screening tools. Importantly, these instruments need to be stable and especially invariant across cultures, thus allowing for cross-cultural comparison. Therefore, this study aimed to describe the translation and cultural adaptation of the CET to Brazilian Portuguese, to test several factor structures among Brazilian and U.S. adults and to examine measurement invariance across countries, and finally, to evaluate the internal consistency of the CET. Furthermore, we sought to test convergent validity, correlating the CET with a measure of eating disorder symptoms, and to compare compulsive exercise symptoms between samples from both countries.

As hypothesized, the three-factor solution with 15 items [22] showed best fit to data. This factor structure proved to be invariant between Brazilians and Americans, and it showed good convergent validity and adequate internal consistency; confirming our hypotheses. In addition, Brazilians demonstrated higher scores on the *Avoidance and rule-driven behavior* and *Mood improvement* subscales, whereas Americans scored higher on the *Weight control exercise* subscale. It is worth noting that no statistically significant difference was observed between countries for the total CET score. This result is contrary to our hypothesis, however the practical significance (i.e., effect size) observed in these comparisons is small.

The original five-factor model proposed by Taranis et al. [14] showed poor fit indices for both samples. This result was expected, as previous validation studies showed that the *Lack of exercise enjoyment* and *Exercise rigidity* subscales are problematic [22, 26, 27, 29]. As previously mentioned, the use of PCA instead of EFA/CFA may potentially explain the differences in fit in our analyses compared to Taranis et al. [14]. Other studies that replicated the original five-factor solution with 24 items also violate some theoretical assumptions [24, 28, 30, 31].

The four-factor solution [26] did not show adequate indices for either sample due to high χ^2^/*df* values and low factor loading on some items. Furthermore, the three-factor solution with 18 items [26] did not show adequate fit indices for the Americans due to high χ^2^/*df* value and low factor loading on item #8. Previous validation studies also found low factor loadings in several items [22, 24, 26, 29, 30]. Despite that, some previous studies [24, 30] chose to keep problematic items.

After carefully analyzing several models, the only model that presented a good fit and adequate factor loadings for each item was the one proposed by Plateau et al. [22]. It is believed that removing the *Lack of exercise enjoyment* and *Exercise rigidity* subscales and the problematic items from the *Avoidance and rule-driven behavior* (items # 11 and 15) and *Mood Improvement* (item # 8) subscales generated a more robust structure for the CET. A similar result can be observed in the study by Rica et al. [27] who tested the three-factor structures for a sample of Spanish men and found a better fit for the model proposed by Plateau et al. [22].

Our findings in no way minimize the quality of the CET as an effective and important assessment tool. In fact, the original validation study of the CET [14] showed that the *Lack of exercise enjoyment* subscale did not correlate with the CET total score. Perhaps lack of exercise enjoyment do not discriminate individuals with and without compulsive exercise. Therefore, this subscale may not add value to the measurement of compulsive exercise. In addition, exercise rigidity seems to be a pattern of relationship with exercise (e.g., commitment to exercise), without necessarily being associated with the compulsive nature of the compulsive exercise. The modified structure of the CET showed good adjustment indices. Thus, it is believed that the CET was developed on a consistent theoretical basis and, in fact, is capable of capturing compulsive traits of dysfunctional exercise. This explains the wide use of the instrument.

We also examined the convergent validity of the CET using the EDE-Q global score as a convergent measure, as was done in previous validation studies [22, 26, 30]. We confirmed previous results, finding correlations between the EDE-Q and CET subscales scores. Specifically, we found small correlation between the EDE-Q and the *Avoidance and rule-driven behavior* subscale among Brazilians and Americans; and a strong correlation between the EDE-Q and the *Weight control exercise* subscale in both samples. A small correlation was observed between the *Mood improvement* subscale and EDE-Q among Americans, but this was non-significant for Brazilians. These results are expected as compulsive exercise is characterized as a compulsive behavior, usually related to body weight and shape concerns and disordered eating/eating disorders [15, 18]. Interestingly, the strongest associations were found between the EDE-Q and the *Weight control exercise* subscale in both samples. Our results provide initial evidence that compulsive exercise may be a relevant risk factor for eating disorders in the Brazilian context. Thus, future studies should assess the sensitivity of the CET in distinguishing participants or patients with small, medium, and high risk for eating disorders.

We found good estimated internal consistency of the CET subscales among Brazilian and American samples. Results are comparable with internal consistencies found in previous validation studies [22, 26, 27].

Previous studies have examined the CET invariance in several contexts, including with different ages, and various eating disorders diagnostic groups [29], as well as in athletes performing at competitive levels [27]. However, the invariance of the CET across different linguistic and cultural contexts had not been explored. Following the recommendation of Swami and Barron [34], we conducted a multigroup CFA to test the CET invariance across Brazilians and Americans. Our results showed the CET's invariance at the configural, metric, and scalar levels, indicating that the instrument presents similar parameters in both cultural contexts. Thereby, authors interested in understanding the cross-cultural differences between the two countries regarding compulsive exercise and correlate thoughts and behaviors now have an instrument with adequate indicators of valid and reliable for this purpose.

In addition to the result found in the invariance analysis, cultural differences must be considered. It should be noted that while there are many similarities among Brazilian and U.S. adults in the value they place on fitness and exercise, there are also important cultural factors within each country that may modulate the way each group values the engagement in exercise and the benefits derived from it. Similarly, cultural differences may relate to the mechanisms through which compulsive exercise coacts with eating pathology to elevate risk. Understanding such differences makes it possible to look at the similarity pointed out by the invariance analysis in a more appropriate way. For example, Forbes et al. [39] found higher scores for body dissatisfaction and lower scores for eating disorders in Brazilian women compared to U.S. women. A study by Carvalho et al. [50] highlights the importance of exercise in both countries yet shows that U.S. men have a higher weekly average of exercises than Brazilian men.

Regarding the comparison of the U.S. and Brazilian samples we found that although there were not significant differences among the groups in CET total score they did vary with higher subscale scores on mood and avoidance in Brazilian adults and higher scores on weight control for U.S. adults. In both samples CET total scores were significantly positively correlated with eating pathology scores. These cultural differences are intriguing and suggest that there are differences in the meaning of exercise among these two cultures. In a previous cross-cultural study by de Carvalho et al. [50] Brazilians were more likely to respond that they exercise for “weight loss” whereas Americans more often said “health.” Perhaps when it comes to compulsive exercise, the higher levels of concern for health among Americans is now reflected in concerns more explicitly for weight loss (given the obesity epidemic in the U.S. today). Perhaps Brazilians who exercise more compulsively find that their moods are affected when they do not or cannot exercise and that now is a stronger motivation that weight loss. In this study we did not examine any psychosocial predictors of compulsive exercise so we are unable to provide empirical evidence to explain these group differences. However, we did show that in both groups higher CET scores related to higher eating pathology and this is consistent with findings from other studies in both clinical and non-clinical samples [22, 26, 30]. Based upon our findings in this study, we found differential risk patterns of compulsive exercise that may be relevant for prevention/intervention efforts with individuals at risk for compulsive exercise and/or pathological eating behaviors.

In conclusion, the Brazilian three-factor-15-item version of the CET solution shows evidence of validity and reliability for Brazilian adults. Thus, the Brazilian CET can be used as a screening measure for dysfunctional exercise. Furthermore, this version was invariant across the Brazilian and American samples; allowing cross-cultural comparison between these countries.

### Strength and limits

The results of the present study are promising in indicating a valid, reliable and invariant factorial structure of the CET between Brazil and the United States. Our findings demonstrate the value of using this measure to assess compulsive exercise in Portuguese-speaking Brazilian adults, and further confirmed its value as an effective measure for English-speaking American adults. Future studies may benefit from cross-cultural comparisons to better understand sociocultural aspects related to compulsive exercise and its role in disordered eating across different cultures.

Although the results found in this study are promising, it does have its limitations. First, the CET was not designed as a diagnostic instrument and thus does not purport to make a clinical diagnosis. However, it will be useful to access compulsive exercise symptoms in Brazilian and American contexts, given elevated risk for dysfunctional exercise in these groups. Second, a nonprobabilistic sample was used, which can limit the generalizability of the present results. Third, assessments were limited to self-report, which may reflect social bias [34]. However, this approach has been used in similar studies [14, 22, 24, 26, 27, 29, 30]. Fourth, differences were observed in the proportions of men and women, as well as individuals diagnosed with eating disorders between the samples from Brazil and the U.S. It is not possible to measure the impact of these sample differences on the data in the present study. It is noteworthy that studies indicate different prevalence of compulsive exercise between men and women [2, 3], as well as high compulsive exercise in individuals with eating disorders [6, 15, 18]. Finally, we used only one measure to investigate the convergent validity of the CET. Future studies should examine the relationship between the CET and other individual variables, such as obsessive–compulsive traits, depressive symptoms, and perfectionism.

### What is already known on this subject?

Compulsive exercise is a compulsive behavior, usually related to body weight and shape concerns, and disordered eating/eating disorders. The Compulsive Exercise Test (CET) is a widely used measure of compulsive exercise, but its factor structure shown to be unstable in several validation studies, which limits conducting cross-cultural studies. Evaluating an adequate factorial structure and its invariance between cultures is of fundamental importance.

### What this study adds?

This study presents important advances in the measurement of compulsive exercise, as well as the relationship between dysfunctional exercise and disordered eating/eating disorders. We tested several factor structures of the CET and its validity, reliability, and measurement invariance across Brazilians and Americans. Results support the CET as a measure of compulsive exercise among Brazilians and Americans, allowing cross-cultural comparisons between these countries.

## Data Availability

The data that support the findings of this study are available from the corresponding author upon reasonable request.
